# Localization of the paranodal protein Caspr in the mammalian retina

**Published:** 2010-09-12

**Authors:** Brendan J. O’Brien, Arlene A. Hirano, Elizabeth D. Buttermore, Manzoor A. Bhat, Elior Peles

**Affiliations:** 1 Research School of Biology, The Australian National University, Canberra, ACT, Australia; 2 Optometry & Vision Science, University of Auckland, Private Bag 92019, Auckland, New Zealand; 3 Department of Neurobiology, Geffen School of Medicine at UCLA, Los Angeles, CA; 4 Curriculum in Neurobiology, Department of Cell and Molecular Physiology, UNC-Neuroscience Center and Neurodevelopmental Disorders Research Center, University of North Carolina School of Medicine, Chapel Hill, NC; 5 Molecular Cell Biology, Weizmann Institute of Science, Rehovot, Israel

## Abstract

**Purpose:**

The retina has the demanding task of encoding all aspects of the visual scene within the space of one fixation period lasting only a few hundred milliseconds. To accomplish this feat, information is encoded in specialized parallel channels and passed on to numerous central nuclei via the optic nerve. These parallel channels achieve specialization in at least three ways: the synaptic networks in which they participate, the neurotransmitter receptors expressed and the types and locations of ion channels or transporters used. Subcellular localization of receptors, channels and transporters is made yet more complex in the retina by the double duty many retinal processes serve. In the present work, we show that the protein Caspr (Contactin Associated Protein), best known for its critical role in the localization of voltage-gated ion channels at the nodes of Ranvier, is present in several types of retinal neurons including amacrine, bipolar, horizontal, and ganglion cells.

**Methods:**

Using standard double label immunofluorescence protocols, we characterized the pattern of Caspr expression in the rodent retina.

**Results:**

Caspr labeling was observed through much of the retina, including horizontal, bipolar, amacrine, and ganglion cells. Among amacrine cells, Caspr was observed in AII amacrine cells through co-localization with Parvalbumin and Disabled-1 in rat and mouse retinas, respectively. An additional amacrine cell type containing Calretinin also co-localized with Caspr, but did not co-localize with choline-acetyltransferase. Nearly all cells in the ganglion cell layer contain Caspr, including both displaced amacrine and ganglion cells. In the outer retina, Caspr was co-localized with PKC labeling in rod bipolar cell dendrites. In addition, Caspr labeling was found inside syntaxin-4 'sandwiches' in the outer plexiform layer, most likely indicating its presence in cone bipolar cell dendrites. Finally, Caspr was co-localized in segments of horizontal cell dendrites labeled with Calbindin-D28k.

**Conclusions:**

Caspr is best known for its role in organizing the localization of different voltage-gated ion channels in and around nodes of Ranvier. As neuronal processes in the retina often play a dual role involving both input and output, it is possible that the localization of Caspr in the retina will help us decipher the way retinal cells localize ion channels in their processes to increase computational capacity.

## Introduction

Until recently, neurons were considered to be polarized structures with passive electrical properties attributed to dendrites, while active properties were the exclusive province of the soma and axon. It is now clear, however, that dendrites in some neurons do indeed have active properties, even generating action potentials (reviewed in [1]). In the retina, the definitions of axon and dendrite are still more blurred, as many neuronal processes serve both functions. How is it then possible for voltage-gated ion channel proteins required for the generation of action potentials to be targeted to the appropriate cellular compartments?

An extensive body of literature regarding this issue has examined the properties of axon initial segments and nodes of Ranvier in retinal ganglion cells. In both cases, it appears that the cytoskeletal binding protein ankyrin-G plays a major role in anchoring voltage-gated sodium channels (VGSCs) at these locations through binding directly [2] or via VGSC β subunits [3]. In contrast, voltage-gated potassium channels (VGKCs) are localized outside nodes, in the juxtaparanode. Between the VGSCs and VGKCs is an area known as the paranode, where septate-like junctions between the axon and myelin sheath are formed. These paranodal axoglial junctions function as an extracellular diffusion barrier and limit lateral diffusion of membrane-associated proteins. One of the key components of the paranodal membrane is Caspr, a single transmembrane protein that helps define the functional subcompartments at nodes [4–10]. The critical role of Caspr in the organization of nodes was demonstrated most directly through generation of a *Caspr* knockout mouse model [5,7]. In *caspr*^−/−^ mutants, VGSCs remained clustered at nodes, whereas VGKCs were more widely distributed in the axon than normal and partially overlapped with VGSCs at the nodes. This resulted in lengthened action potential generation, reduced conduction velocity and other serious neuromuscular disturbances, eventuating in death [5]. Thus, the role of Caspr in the organization of molecular domains in myelinated axons is critical for information processing in the nervous system.

Given the dual functional roles of axo-dendritic retinal processes, we wondered whether Caspr might play a role in the localization of voltage-gated sodium and potassium channels in the retina. Previous studies of retinal Caspr expression suggest that it was limited to retinal ganglion cell (RGC) somas and axons, with some diffuse labeling in the inner plexiform layer (ipl; [4,11]). Our data demonstrate that Caspr is expressed not only by RGCs, but also several types of amacrine cell, horizontal cells and bipolar cells in the rodent retina. Every neural cell class in the retina has been previously demonstrated as having the capacity to generate action potentials (e.g., photoreceptors [12], bipolar cells [13], horizontal cells [14], and amacrine cells [15]). Thus, Caspr may indeed play similar roles in both the retina and optic nerves.

## Methods

### Animals

All animal experiments were performed according to guidelines for ethical treatment of laboratory animals, as outlined by the Society for Neuroscience, and were approved by the University of Auckland Animal Ethics Committee, the Australian National University Animal Experimentation Ethics Committee, and Institutional Animal Care and Use Committees for both the University of California, Los Angeles and the University of North Carolina.

### Tissue preparation and immunohistochemistry

Detailed descriptions of techniques can be found in Hirano et al. (2007) and O'Brien et al. (2008). In brief, animals were anaesthetized and the eyes of mice and rats were enucleated, hemisected and the remaining eyecups fixed in 4% paraformaldehyde for variable times (15, 30, and 60 min). The data presented in this paper come from mice (n=16) and rats (n=9). After washing thoroughly in phosphate buffered saline (PBS, pH=7.3), eyecups were placed in 30% sucrose in PBS for cryoprotection, sectioned on a cryostat at 14–18 μm and mounted onto Superfrost plus slides. Primary antibodies (see Table 1) were applied to the tissue sections in a solution containing 5% Chemiblocker (Chemicon) and 0.5% Triton X-100 (Sigma) overnight at room temperature. After washing thoroughly with PBS, species-appropriate secondary antibodies conjugated to Alexa 488 or Alexa 594 (Invitrogen) were applied in the same solution for 1–2 h at room temperature, while protected from light exposure. Alternatively, retinal wholemounts were immunostained in the same fashion using longer incubation times (primary antibody applied for 3 days and secondary antibodies applied overnight). After a final series of washes, immunostained sections or wholemounts were coverslipped with Aqua polymount (Polysciences) and imaged using a fluorescence photomicroscope (Leica) or using a Zeiss PASCAL or LSM510 confocal laser scanning microscope with a 40x C-Apochromat 1.2 NA water objective. Acquired images were imported into Adobe Photoshop CS for global application of filters to enhance brightness and/or contrast.

**Table 1:** List of antibodies used in this study.

**Antibody against:**	**Host**	**Dilution**	**Immunogen**	**Specificity**	**Reference**	**Source**	**Cat no.**	**Rodent retina**
CASPR monoclonal	mouse	1:200	C-terminus	Knockout/western	Figure 1	Peles	–	Figure 1, [54]
CASPR polyclonal	rabbit	1:200	Cytoplasmic	Knockout/western	[4]	Peles	–	see Figure 1 and Figure 3
Neurofilament 200	mouse	1:200	C-terminus	Western 200 kDa	[55]	Sigma	N0142	[29]
ChAT	rabbit	1:200	full length	immunohistochemistry	Manufacturers product desc.	Millipore	AB143	[56]
Calretinin	rabbit	1 :4000	full length	Western 29 kDa	[57]	Swant	7699/4	[28]
Parvalbumin	rabbit	1:1000	full length	Western 12 kDa	[58]	Swant	PV28	[21]
Calbindin D28k	rabbit	1:1000	full length	Immunoblot 28 kDa	[59]	Swant	CB38	[28]
Disabled-1	rabbit	1:1000	C-terminus	Knockout/western	Manuf. Product Desc	Rockland	100–401–225	[20]
Protein Kinase C	mouse	1:1000	full length	Epitope mapping/western	[60]	Biodesign	K01107M	[26]
Syntaxin 4	rabbit	1:400	N-terminus	Knockout/western	[61,62]	Millipore	AB5330	[26]

Immunogen is given as specifically as is known. Antibody specificity has been tested in our laboratories (Caspr) or by the techniques listed in the associated reference. Prior studies using these antibodies upon rodent retina and optic nerve are also cited.

To demonstrate specificity of retinal Caspr labeling, we performed immunostaining of retinal tissue obtained from *Caspr* knockout mice [5] by applying both monoclonal and polyclonal antibodies to Caspr. No labeling was observed for either antibody upon retinal tissue from *Caspr* knockout animals (see Results for further description).

## Results

### Localization of Caspr in rat and mouse retina

**Figure 1:**
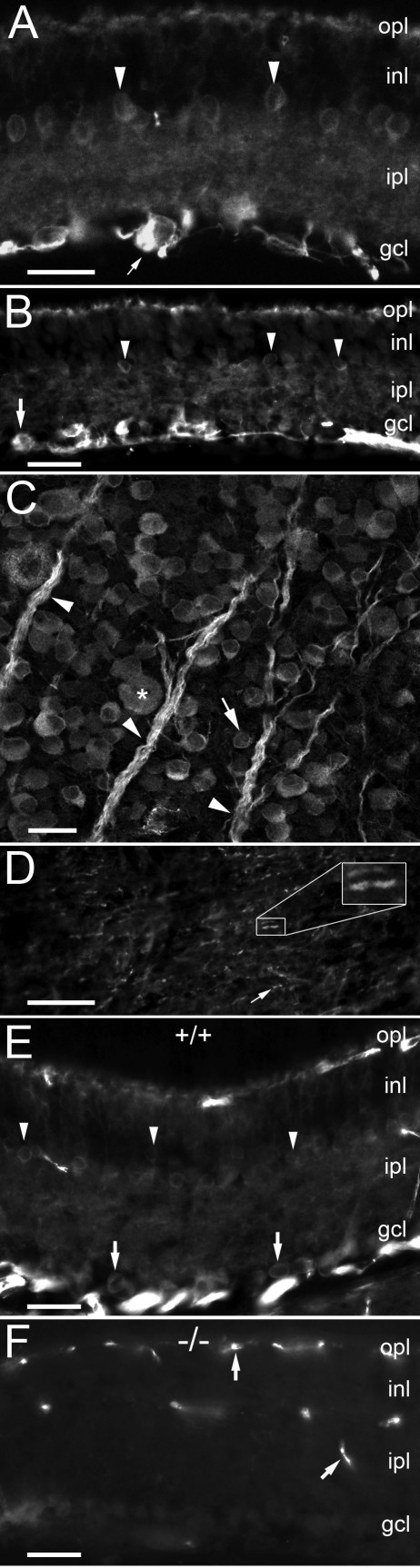
Caspr labeling in rat and mouse retina. **A**: Photomicrograph of monoclonal Caspr labeling in rat retina. Arrow indicates one of several retinal ganglion cells (RGCs) intensely labeled by Caspr. In addition to RGCs, somas of many amacrine cells in the inner nuclear layer (inl) were also labeled (e.g., arrowheads). The inner plexiform layer (ipl) was diffusely labeled while the outer plexiform layer (opl) contained several hot spots. **B**: Lower power photomicrograph of Caspr labeling in rat retina using a rabbit polyclonal antibody. A nearly identical pattern of labeling was observed as in **A**. **C**: Single confocal section of rat retinal wholemount labeled with a monoclonal Caspr antibody. Intense labeling of axon fiber bundles was observed (arrowheads) as well as somas of nearly all cells in the ganglion cell layer. Both large RGCs (asterisk) and likely displaced amacrine cells (arrow, somas <10 μm) were labeled. **D**: Photomicrograph of Caspr labeling in the rat optic nerve. Inset shows magnified view of node indicated. Arrow indicates another labeled node. **E**: Photomicrograph of Caspr labeling (mAb) in mouse retina. Similar to the rat retina (**A**, **B**) intense labeling of RGCs and fiber bundles were observed as well as many amacrine cells in the inl. **F**: Photomicrograph of Caspr labeling (mAb) in knockout retina. All labeling of retinal cell types was eliminated, leaving only nonspecific labeling of retinal blood vessels. Scale bars equal to 25 μm.

As was expected for Caspr, we observed very intense labeling of retinal ganglion cell somas and their axons in radial sections of rat retina (e.g., arrows Figure 1A,B [4,11]). Surprisingly, we also observed additional, previously unreported labeling of somata in the inner nuclear layer (inl). Most of these labeled somas (arrowheads, Figure 1A,B) were observed at the boundary between the inl and inner plexiform layers (ipl) of the retina, indicating their likely classification as amacrine cells. Intense and somewhat patchy Caspr labeling was also found in the outer plexiform layer (opl), while the inner plexiform layer exhibited mostly diffuse labeling. These results were consistent when using either monoclonal (Figure 1A) or polyclonal (Figure 1B) antibodies with Caspr.

In rat retinal wholemount material labeled for Caspr, we observed brilliant labeling of fiber bundles (arrowheads, Figure 1C) and individual axonal segments as well as RGC somas (e.g., asterisk, Figure 1C). Most cells in the ganglion cell layer (gcl) were labeled with Caspr antibodies, including likely displaced amacrine cells with very small soma diameters (8–10 μm; e.g., arrow, Figure 1C).

To demonstrate that this pattern of Caspr labeling in the retina was not spurious, we also labeled sections of rat optic nerve where Caspr has been previously observed by numerous investigators [4,5,8–11,16–19]. As expected, Caspr labeling in the rat optic nerve included the paranodal regions of nodes of Ranvier (arrow and inset, Figure 1D). As an additional control and comparative study, we labeled wild-type (Figure 1E) and Caspr knockout mouse retina with antibodies to Caspr. The overall pattern of Caspr labeling in the mouse retina was similar to that found in the rat (c.f. Figure 1A,E). This included brilliant labeling of axonal bundles as well as RGC somas (arrows, Figure 1E), less intense labeling of likely amacrine cells in the inl and strong labeling in the opl. Some artifactual labeling of blood vessels was also observed in both wild type and knockout retinal sections. Specificity of the Caspr labeling was evident by comparing this pattern of staining with that found in sections of Caspr knockout mouse retina (Figure 1F). All cellular and fiber labeling for Caspr was absent in retinas of knockout mice, with the exception of blood vessels (Figure 1F).

### Co-localization of Caspr with amacrine cell markers

**Figure 2:**
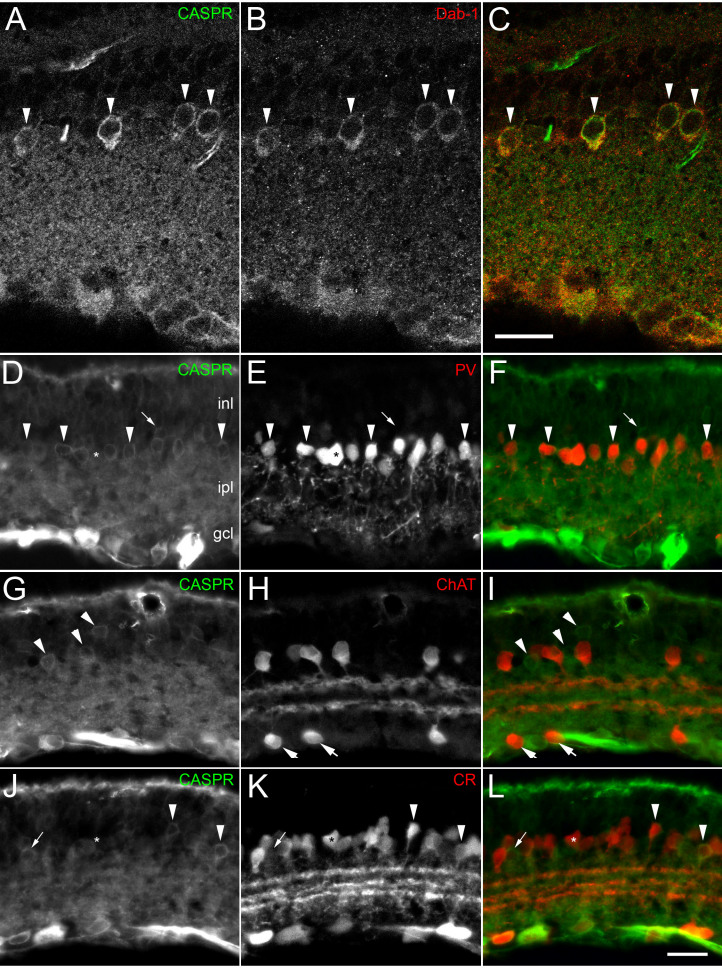
Co-localization of Caspr and inl cell markers in rodent retina. **A**-**C**: Single confocal section demonstrating co-localization of Caspr labeled amacrine cells near the inner nuclear layer/inner plexiform layer (inl/ipl) boundary (**A**, arrowheads) with Disabled-1 (**B**) a marker of AII amacrine cells in mouse retina (**C**, overlay) **D**-**F**: Photomicrographs of rat retina demonstrating that nearly all Caspr labeled amacrine cells (e.g., arrowheads, **D**) also contained Parvalbumin (PV, **E**), a well known marker for AII amacrine cells (**F**, overlay). Arrow in (**D**) indicates a cell labeled with Caspr, but not co-localized with Parvalbumin. Asterisk in (**E**) indicates a cell labeled with Parvalbumin, but not co-localized with Caspr. **G-I**: Photomicrographs of rat retina demonstrating that Caspr (**G**) was not co-localized with ChAT (**H**) in amacrine cells (**I**, overlay). Arrowheads indicate Caspr labeled cells in the inl; arrows indicate ChAT labeled cells in the GCL. **J-L**: Photomicrographs of rat retina showing that some Caspr-labeled amacrine cells (arrowheads, **J**) contained Calretinin (**K** arrowheads, **L** overlay). Arrows in **J**-**L** indicate a Caspr labeled amacrine cell that did not co-localize with Calretinin, while asterisks (**J**-**L**) indicate a Calretinin positive cell that did not contain Caspr. Scale bar equal to 25 μm.

To determine which amacrine cell types were labeled with Caspr, we performed double-label immunohistochemistry experiments with Caspr and well known amacrine cell markers (see Table 1). As the Caspr-labeled cells in the inl were quite numerous and most them were located very close to the inl/ipl border (Figure 2A,D), we suspected they might be AII amacrine cells. AII amacrine cells in the mouse retina are well known to be labeled by antibodies to Disabled-1 (Dab-1 [20]). Clear double labeling of Caspr with Dab-1 (Figure 2A-C) was observed. Similarly, we observed that Caspr labeling in the rat inl was co-localized with Parvalbumin (Figure 2D-F), a well known marker of AII amacrine cells in this species [21]. In rat retina, however, some Caspr-positive cells in the inl did not co-localize with Parvalbumin (e.g., arrow, Figure 2D,E), suggesting that there are at least two cell types labeled with Caspr in the inl.

Since Caspr antibodies labeled at least one other cell type in the inl aside from AII amacrine cells, we examined whether this might be a cholinergic amacrine cell (Figure 2G-I). Double labeling experiments with Caspr (Figure 2G) and choline acetyltransferase (ChAT, Figure 2H) demonstrated no co-localization in either the inl (arrowheads, Figure 2G,I) or the gcl (Figure 2H,I; arrows).

Another calcium binding protein, Calretinin, has been previously shown to label both amacrine and ganglion cells in rat retina [22,23]. Previous double labeling studies have demonstrated that cholinergic amacrine cells and at least one other amacrine cell type contain Calretinin [24,25]. Double labeling experiments of Calretinin with Caspr demonstrated that a small minority of Calretinin positive amacrine cells, localized closer to the middle of the ipl, also labeled with Caspr (arrowheads, Figure 2J-L). Since AII amacrine cells do not contain Calretinin, most Caspr positive cell somas near the inl/ipl border were not double labeled (e.g., arrow, Figure 2J,K). Thus, Caspr is present in at least three different types of amacrine cells (displaced, AII and a rare Calretinin positive type), but is not present in cholinergic amacrine cells.

### Caspr labeling in the outer plexiform layer

**Figure 3:**
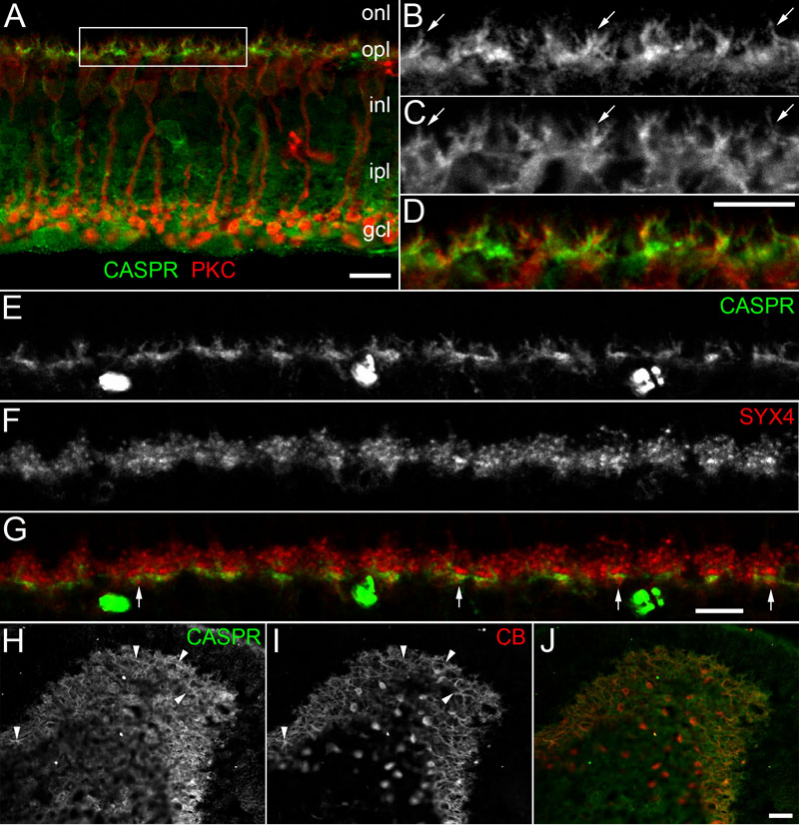
Localization of Caspr in the outer plexiform layer. **A**: Immunostaining for Caspr (green) labeled an intense band in the outer plexiform layer (opl) as well as AII amacrine cells in the inner nuclear layer (inl) of mouse retina (Scale bar equal to10 μm). Double labeling with PKC (red) demonstrated co-localization with Caspr in the opl. Projection of 5 images totaling a stack thickness of 1.2 µm. **B-D**: Higher magnification images of Caspr (**B**), PKC (**C**) and overlay (**D**) of outlined region in **A**. Arrows in **B**, **C** indicate double labeled rod bipolar cell dendrites in the opl. Scale bar in **D** equal to 10 μm. **E**-**G**: High power images of double labeling for Caspr (**E**), Syx4 (**F**) and overlay in opl of mouse retina. Syx4 labels horizontal cell tips and densely labeled ‘sandwiches’ postsynaptic to cone pedicles (arrows, **G**). Note that Caspr labeling is largely not co-localized, and in several cases falls within a Syx4 sandwich. Large spots of labeling below opl in **E** are nonspecifically labeled blood vessels. Scale bar in **G** equal to 10 μm. Projection of 5 images totaling a stack thickness of 1.2 µm. **H-J**: Double labeling of Caspr (**H**) with Calbindin (**I**) and overlay (**J**) in tangential section through the opl in mouse retina. Arrowheads indicate horizontal cell processes double labeled with Caspr (**H**) and Calbindin (**I**). Scale bar in **J** equal to 25 μm.

In addition to labeling the inner retina, Caspr antibodies also labeled the outer plexiform layer (opl, Figure 3A). In radial sections through the retina, the opl appeared intensely labeled with several identifiable protrusions from the opl into the outer nuclear layer (Figure 3B,C; onl, arrows). The cellular identity of these protrusions, however, was unclear. Co-immunostaining of Caspr with protein kinase C antibodies (PKC; Figure 3A-D) revealed extensive co-localization in the dendrites of rod bipolar cells. Interestingly, Caspr seemed to be co-localized with PKC in proximal parts of bipolar cell dendrites, but was not observed in the extreme dendritic tips.

From the Caspr labeling we observed in the opl, it appeared as if horizontally oriented elements were also present close to the opl/inl boundary. We therefore investigated whether horizontal cell dendrites might also be labeled. To examine this issue, we co-immunostained mouse retina with antibodies to Caspr (Figure 3E) and syntaxin-4 (Syx4, Figure 3F, overlay Figure 3G), which is a marker for horizontal cell dendritic tips in mammalian retina [26]. Similar to Figure 3B, the Caspr labeling consisted of intense labeling in the opl with short protrusions into the onl. Interestingly, the intense, more horizontally oriented Caspr labeling (Figure 3E,G) appeared at a semi-regular interval, and when compared with the Syx4 labeling (Figure 3F,G), it was localized near the opl/inl boundary. Essentially no co-localization of Caspr and Syx4 was observed at the tips of horizontal cell dendrites. Instead, it appeared as if Caspr labeling was in many cases interdigitated with labeling for Syx4 (arrows Figure 3G) near the opl/inl boundary. Syntaxin-4 “sandwiches” have been previously characterized in mammalian retina as processes of horizontal cells postsynaptic to cone axon terminals [26]. Our data suggest that the intense, horizontally oriented Caspr labeling in the opl is found inside the Syx4 sandwiches in many cases (arrows, Figure 3G), and is therefore likely to be labeling proximal dendrites of cone bipolar cells [27], similar to the labeling we have observed for rod bipolar cells.

To look more closely at whether Caspr might be present at other locations in horizontal cell dendrites, we also double labeled sections with Caspr (Figure 3H,J) and Calbindin-D28k (Figures 3I,J) a well known marker for horizontal cells in mouse and rat retina [26,28]. Our data demonstrated that most of horizontal cell processes were not double labeled. On occasion, however, horizontal cell dendrites were observed to be double labeled (arrowheads, Figures 3H,I). Double labeling of Caspr and an antibody to Neurofilament 200 was used to label horizontal cell axons [29], but no co-localization was demonstrated (data not shown).

## Discussion

Our data demonstrate that the well known paranodal protein Caspr is present in four of the five neuronal cell types in the retina, including retinal ganglion cells, amacrine cells, bipolar cells and horizontal cells. While the role of Caspr in RGC axons in the optic nerve has been extensively studied [4,5,8–11,16–19], the functional role of Caspr inside the retina is unclear. In the optic nerve, it is believed that Caspr plays a significant role in the segregation of voltage-gated sodium and potassium channels in nodes of Ranvier and juxtaparanodes, respectively [30]. In *caspr^−/−^* mutant mice, action potentials are lengthened and conduction velocity significantly slowed in nerve fibers [5]. These data suggest that Caspr’s role in determining the precise localization of voltage-gated ion channels in cellular membranes plays a significant role in the propagation of action potentials. Is it possible that Caspr plays similar roles both inside the retina and in the optic nerve?

Inside the retina, the role of action potentials is controversial. While it has been demonstrated that all retinal cell classes possess the capacity to generate at least one action potential, only a subset of these are capable of repetitive spiking. While most, if not all, RGC types have the capacity for repetitive spiking [31–35], only some amacrine cell types and rabbit horizontal cells have been demonstrated to generate repetitive spiking [14,15,36–43]. It is interesting to note that Caspr is expressed in a similar pattern, being found in nearly all cells in the ganglion cell layer (including both RGCs and many displaced amacrine cells), AII amacrine cells and horizontal cells. Oddly, however, we only found strong evidence for the expression of Caspr in amacrine cell somata, bipolar cell dendrites, and the somas and unmyelinated axons of RGCs inside the retina. There did not appear to be obvious labeling of processes in the ipl, and while Caspr was found in horizontal cell dendrites, it did not appear to co-localize with Neurofilament 200 or Syntaxin-4 in their axons (data not shown). This apparent paradox may be due simply to the fixation parameters used, or perhaps to different post-translational modifications (e.g., phosphorylation or binding partners) present in different parts of the cell.

While it has been demonstrated that the remaining classes of retinal neurons (photoreceptors, bipolar and horizontal cells) have the capacity to generate sodium action potentials [12–14,44,45], whether they do so in situ in the retina is yet unclear. What is clear from these and a host of other studies, however, is that all five classes of retinal neuron do express voltage-gated sodium and potassium channels. These channels can provide additional computational capacity to a neuron beyond just the generation of spikes. For example, voltage-gated sodium channels can enter an alternate conductance state and generate persistent inward sodium currents. These currents have been implicated in generating rhythmicity, boosting synaptic inputs in dendrites and can be activated by neurotransmitters via G proteins [46,47]. Persistent sodium currents and their associated channels have been described previously in various retinal cell types [36,48–53]. The broad expression of Caspr in the retina described here may therefore help us to decipher how retinal cells localize ion channels to increase their computational capacity. Further anatomic and physiologic study of *caspr*^−/−^ mutants will be required to determine the functional role(s) played by Caspr in the retina.

## References

[1] Gulledge AT Kampa BM Stuart GJ Synaptic integration in dendritic trees. J Neurobiol 2005 64 75 90 15884003 10.1002/neu.20144

[2] Bréchet A Fache MP Brachet A Ferracci G Baude A Irondelle M Pereira S Leterrier C Dargent B Protein kinase CK2 contributes to the organization of sodium channels in axonal membranes by regulating their interactions with ankyrin G. J Cell Biol 2008 183 1101 14 19064667 10.1083/jcb.200805169 PMC2600743

[3] Malhotra JD Kazen-Gillespie K Hortsch M Isom LL Sodium channel beta subunits mediate homophilic cell adhesion and recruit ankyrin to points of cell-cell contact. J Biol Chem 2000 275 11383 8 10753953 10.1074/jbc.275.15.11383

[4] Peles E Nativ M Lustig M Grumet M Schilling J Martinez R Plowman GD Schlessinger J Identification of a novel contactin-associated transmembrane receptor with multiple domains implicated in protein-protein interactions. EMBO J 1997 16 978 88 9118959 10.1093/emboj/16.5.978 PMC1169698

[5] Bhat MA Rios JC Lu Y Garcia-Fresco GP Ching W St Martin M Li J Einheber S Chesler M Rosenbluth J Salzer JL Bellen HJ Axon-glia interactions and the domain organization of myelinated axons requires neurexin IV/Caspr/Paranodin. Neuron 2001 30 369 83 11395000 10.1016/s0896-6273(01)00294-x

[6] Rios JC Rubin M St Martin M Downey RT Einheber S Rosenbluth J Levinson SR Bhat M Salzer JL Paranodal interactions regulate expression of sodium channel subtypes and provide a diffusion barrier for the node of Ranvier. J Neurosci 2003 23 7001 11 12904461 10.1523/JNEUROSCI.23-18-07001.2003 PMC6740666

[7] Gollan L Salomon D Salzer JL Peles E Caspr regulates the processing of contactin and inhibits its binding to neurofascin. J Cell Biol 2003 163 1213 8 14676309 10.1083/jcb.200309147 PMC2173730

[8] Gollan L Sabanay H Poliak S Berglund EO Ranscht B Peles E Retention of a cell adhesion complex at the paranodal junction requires the cytoplasmic region of Caspr. J Cell Biol 2002 157 1247 56 12082082 10.1083/jcb.200203050 PMC2173544

[9] Rasband MN Peles E Trimmer JS Levinson SR Lux SE Shrager P Dependence of nodal sodium channel clustering on paranodal axoglial contact in the developing CNS. J Neurosci 1999 19 7516 28 10460258 10.1523/JNEUROSCI.19-17-07516.1999 PMC6782503

[10] Hinman JD Peters A Cabral H Rosene DL Hollander W Rasband MN Abraham CR Age-related molecular reorganization at the node of Ranvier. J Comp Neurol 2006 495 351 62 16485288 10.1002/cne.20886 PMC4444368

[11] Boiko T Rasband MN Levinson SR Caldwell JH Mandel G Trimmer JS Matthews G Compact myelin dictates the differential targeting of two sodium channel isoforms in the same axon. Neuron 2001 30 91 104 11343647 10.1016/s0896-6273(01)00265-3

[12] Kawai F Horiguchi M Suzuki H Miyachi E Na(+) action potentials in human photoreceptors. Neuron 2001 30 451 8 11395006 10.1016/s0896-6273(01)00299-9

[13] Pan ZH Hu HJ Voltage-dependent Na(+) currents in mammalian retinal cone bipolar cells. J Neurophysiol 2000 84 2564 71 11067998 10.1152/jn.2000.84.5.2564

[14] Blanco R Vaquero CF de la Villa P Action potentials in axonless horizontal cells isolated from the rabbit retina. Neurosci Lett 1996 203 57 60 8742046 10.1016/0304-3940(95)12263-x

[15] Boos R Schneider H Wässle H Voltage- and transmitter-gated currents of all-amacrine cells in a slice preparation of the rat retina. J Neurosci 1993 13 2874 88 7687279 10.1523/JNEUROSCI.13-07-02874.1993 PMC6576675

[16] Caldwell JH Schaller KL Lasher RS Peles E Levinson SR Sodium channel Na(v)1.6 is localized at nodes of ranvier, dendrites, and synapses. Proc Natl Acad Sci USA 2000 97 5616 20 10779552 10.1073/pnas.090034797 PMC25877

[17] Van Wart A Matthews G Impaired firing and cell-specific compensation in neurons lacking nav1.6 sodium channels. J Neurosci 2006 26 7172 80 16822974 10.1523/JNEUROSCI.1101-06.2006 PMC6673932

[18] Chen C Westenbroek RE Xu X Edwards CA Sorenson DR Chen Y McEwen DP O'Malley HA Bharucha V Meadows LS Knudsen GA Vilaythong A Noebels JL Saunders TL Scheuer T Shrager P Catterall WA Isom LL Mice lacking sodium channel beta1 subunits display defects in neuronal excitability, sodium channel expression, and nodal architecture. J Neurosci 2004 24 4030 42 15102918 10.1523/JNEUROSCI.4139-03.2004 PMC6729427

[19] Rasband MN Trimmer JS Peles E Levinson SR Shrager PK + channel distribution and clustering in developing and hypomyelinated axons of the optic nerve. J Neurocytol 1999 28 319 31 10739574 10.1023/a:1007057512576

[20] Rice DS Curran T Disabled-1 is expressed in type AII amacrine cells in the mouse retina. J Comp Neurol 2000 424 327 38 10906706 10.1002/1096-9861(20000821)424:2<327::aid-cne10>3.0.co;2-6

[21] Wässle H Grünert U Rohrenbeck J Immunocytochemical staining of AII-amacrine cells in the rat retina with antibodies against parvalbumin. J Comp Neurol 1993 332 407 20 8349840 10.1002/cne.903320403

[22] Veruki ML Wässle H Immunohistochemistry localization of dopamine D1 receptors in rat retina. Eur J Neurosci 1996 8 2286 97 8950093 10.1111/j.1460-9568.1996.tb01192.x

[23] Pasteels B Rogers J Blachier F Pochet R Calbindin and calretinin localization in retina from different species. Vis Neurosci 1990 5 1 16 2125465 10.1017/s0952523800000031

[24] Araki CM Hamassaki-Britto DE Calretinin co-localizes with the NMDA receptor subunit NR1 in cholinergic amacrine cells of the rat retina. Brain Res 2000 869 220 4 10865078 10.1016/s0006-8993(00)02364-7

[25] Gábriel R Witkovsky P Cholinergic, but not the rod pathway-related glycinergic (All), amacrine cells contain calretinin in the rat retina. Neurosci Lett 1998 247 179 82 9655622 10.1016/s0304-3940(98)00323-1

[26] Hirano AA Brandstätter JH Vila A Brecha NC Robust syntaxin-4 immunoreactivity in mammalian horizontal cell processes. Vis Neurosci 2007 24 489 502 17640443 10.1017/S0952523807070198 PMC2744743

[27] Haverkamp S Grünert U Wässle H The cone pedicle, a complex synapse in the retina. Neuron 2000 27 85 95 10939333 10.1016/s0896-6273(00)00011-8

[28] Haverkamp S Wässle H Immunocytochemical analysis of the mouse retina. J Comp Neurol 2000 424 1 23 10888735

[29] Wässle H Peichl L Airaksinen MS Meyer M Calcium-binding proteins in the retina of a calbindin-null mutant mouse. Cell Tissue Res 1998 292 211 8 9560464 10.1007/s004410051052

[30] Peles E Salzer JL Molecular domains of myelinated axons. Curr Opin Neurobiol 2000 10 558 65 11084317 10.1016/s0959-4388(00)00122-7

[31] O'Brien BJ Isayama T Richardson R Berson DM Intrinsic physiological properties of cat retinal ganglion cells. J Physiol 2002 538 787 802 11826165 10.1113/jphysiol.2001.013009 PMC2290089

[32] Skaliora I Scobey RP Chalupa LM Prenatal development of excitability in cat retinal ganglion cells: action potentials and sodium currents. J Neurosci 1993 13 313 23 8423477 10.1523/JNEUROSCI.13-01-00313.1993 PMC6576296

[33] Sheasby BW Fohlmeister JF Impulse encoding across the dendritic morphologies of retinal ganglion cells. J Neurophysiol 1999 81 1685 98 10200204 10.1152/jn.1999.81.4.1685

[34] Wang GY Ratto G Bisti S Chalupa LM Functional development of intrinsic properties in ganglion cells of the mammalian retina. J Neurophysiol 1997 78 2895 903 9405510 10.1152/jn.1997.78.6.2895

[35] Fohlmeister JF Miller RF Mechanisms by which cell geometry controls repetitive impulse firing in retinal ganglion cells. J Neurophysiol 1997 78 1948 64 9325363 10.1152/jn.1997.78.4.1948

[36] Feigenspan A Gustincich S Bean BP Raviola E Spontaneous activity of solitary dopaminergic cells of the retina. J Neurosci 1998 18 6776 89 9712649 10.1523/JNEUROSCI.18-17-06776.1998 PMC6792954

[37] Lasater EM Dowling JE Ripps H Pharmacological properties of isolated horizontal and bipolar cells from the skate retina. J Neurosci 1984 4 1966 75 6147395 10.1523/JNEUROSCI.04-08-01966.1984 PMC6564952

[38] Heflin SJ Cook PB Narrow and wide field amacrine cells fire action potentials in response to depolarization and light stimulation. Vis Neurosci 2007 24 197 206 17640411 10.1017/S095252380707040X

[39] Bloomfield SA Volgyi B Response properties of a unique subtype of wide-field amacrine cell in the rabbit retina. Vis Neurosci 2007 24 459 69 17900375 10.1017/S0952523807070071

[40] Völgyi B Xin D Amarillo Y Bloomfield SA Morphology and physiology of the polyaxonal amacrine cells in the rabbit retina. J Comp Neurol 2001 440 109 25 11745611 10.1002/cne.1373

[41] Freed MA Pflug R Kolb H Nelson R ON-OFF amacrine cells in cat retina. J Comp Neurol 1996 364 556 66 8820883 10.1002/(SICI)1096-9861(19960115)364:3<556::AID-CNE12>3.0.CO;2-N

[42] Stafford DK Dacey DM Physiology of the A1 amacrine: a spiking, axon-bearing interneuron of the macaque monkey retina. Vis Neurosci 1997 14 507 22 9194317 10.1017/s0952523800012165

[43] Veruki ML Hartveit E AII (Rod) amacrine cells form a network of electrically coupled interneurons in the mammalian retina. Neuron 2002 33 935 46 11906699 10.1016/s0896-6273(02)00609-8

[44] Kawai F Horiguchi M Ichinose H Ohkuma M Isobe R Miyachi E Suppression by an h current of spontaneous Na+ action potentials in human cone and rod photoreceptors. Invest Ophthalmol Vis Sci 2005 46 390 7 15623800 10.1167/iovs.04-0724

[45] Ma YP Cui J Pan ZH Heterogeneous expression of voltage-dependent Na+ and K+ channels in mammalian retinal bipolar cells. Vis Neurosci 2005 22 119 33 15935105 10.1017/S0952523805222010

[46] Crill WE Persistent sodium current in mammalian central neurons. Annu Rev Physiol 1996 58 349 62 8815799 10.1146/annurev.ph.58.030196.002025

[47] Delmas P Raggenbass M Gola M Low-threshold Na+ currents: a new family of receptor-operated inward currents in mammalian nerve cells. Brain Res Brain Res Rev 1997 25 246 54 9403140 10.1016/s0165-0173(97)00022-2

[48] O'Brien BJ Caldwell JH Ehring GR Bumsted O'Brien KM Luo S Levinson SR Tetrodotoxin-resistant voltage-gated sodium channels Na(v)1.8 and Na(v)1.9 are expressed in the retina. J Comp Neurol 2008 508 940 51 18399542 10.1002/cne.21701

[49] Shingai R Christensen BN Sodium and calcium currents measured in isolated catfish horizontal cells under voltage clamp. Neuroscience 1983 10 893 7 6316203 10.1016/0306-4522(83)90227-0

[50] Steffen MA Seay CA Amini B Cai Y Feigenspan A Baxter DA Marshak DW Spontaneous activity of dopaminergic retinal neurons. Biophys J 2003 85 2158 69 14507682 10.1016/s0006-3495(03)74642-6 PMC1303443

[51] Koizumi A Watanabe SI Kaneko A Persistent Na+ current and Ca2+ current boost graded depolarization of rat retinal amacrine cells in culture. J Neurophysiol 2001 86 1006 16 11495968 10.1152/jn.2001.86.2.1006

[52] Margolis DJ Detwiler PB Different mechanisms generate maintained activity in ON and OFF retinal ganglion cells. J Neurosci 2007 27 5994 6005 17537971 10.1523/JNEUROSCI.0130-07.2007 PMC3136104

[53] Hidaka S Ishida AT Voltage-gated Na+ current availability after step- and spike-shaped conditioning depolarizations of retinal ganglion cells. Pflugers Arch 1998 436 497 508 9683721 10.1007/s004240050664

[54] Poliak S Gollan L Martinez R Custer A Einheber S Salzer JL Trimmer JS Shrager P Peles E Caspr2, a new member of the neurexin superfamily, is localized at the juxtaparanodes of myelinated axons and associates with K+ channels. Neuron 1999 24 1037 47 10624965 10.1016/s0896-6273(00)81049-1

[55] Debus E Weber K Osborn M Monoclonal antibodies specific for glial fibrillary acidic (GFA) protein and for each of the neurofilament triplet polypeptides. Differentiation 1983 25 193 203 6198232 10.1111/j.1432-0436.1984.tb01355.x

[56] Jeon CJ Strettoi E Masland RH The major cell populations of the mouse retina. J Neurosci 1998 18 8936 46 9786999 10.1523/JNEUROSCI.18-21-08936.1998 PMC6793518

[57] Schwaller B Buchwald P Blumcke I Celio MR Hunziker W Characterization of a polyclonal antiserum against the purified human recombinant calcium binding protein calretinin. Cell Calcium 1993 14 639 48 8242719 10.1016/0143-4160(93)90089-o

[58] Kägi U Berchtold MW Heizmann CW Ca2+-binding parvalbumin in rat testis. Characterization, localization, and expression during development. J Biol Chem 1987 262 7314 20 3294830

[59] Celio MR Baier W Scharer L Gregersen HJ de Viragh PA Norman AW Monoclonal antibodies directed against the calcium binding protein Calbindin D-28k. Cell Calcium 1990 11 599 602 2285928 10.1016/0143-4160(90)90014-l

[60] Young S Rothbard J Parker PJ A monoclonal antibody recognising the site of limited proteolysis of protein kinase C. Inhibition of down-regulation in vivo. Eur J Biochem 1988 173 247 52 2451608 10.1111/j.1432-1033.1988.tb13991.x

[61] Spurlin BA Thurmond DC Syntaxin 4 facilitates biphasic glucose-stimulated insulin secretion from pancreatic beta-cells. Mol Endocrinol 2006 20 183 93 16099818 10.1210/me.2005-0157

[62] Sherry DM Mitchell R Standifer KM du Plessis B Distribution of plasma membrane-associated syntaxins 1 through 4 indicates distinct trafficking functions in the synaptic layers of the mouse retina. BMC Neurosci 2006 7 54 16839421 10.1186/1471-2202-7-54 PMC1555595

